# Combined Exoscopic and Endoscopic Two-Step Keyhole Approach for Intracranial Meningiomas

**DOI:** 10.3390/curroncol29080426

**Published:** 2022-07-29

**Authors:** Tadashi Watanabe, Kenichiro Iwami, Yugo Kishida, Tetsuya Nagatani, Hiroshi Yatsuya, Shigeru Miyachi

**Affiliations:** 1 Department of Neurosurgery, Aichi Medical University, Nagakute 480-1195, Japan; iwami.kenichirou.371@mail.aichi-med-u.ac.jp (K.I.); miyachi.shigeru.752@mail.aichi-med-u.ac.jp (S.M.); 2 Department of Neurosurgery, Japanese Red Cross Nagoya Daini Hospital, Nagoya 466-8650, Japan; yugok@nagoya2.jrc.or.jp (Y.K.); nagatani4137@nagoya2.jrc.or.jp (T.N.); 3 Department of Public Health and Health Systems, Graduate School of Medicine, Nagoya University, Nagoya 466-8550, Japan; h828@med.nagoya-u.ac.jp

**Keywords:** endoscope, exoscope, meningioma, keyhole approach

## Abstract

The advantages of neuroendoscopic surgery are the wide viewing angle and the freedom of an axis of view with minimal surgical trauma. With the advent of the exoscope, which has similar advantages to endoscopy, such as a small body and ergonomically superior heads-up surgery, it has become possible to add a field of view that is similar to that of microsurgery to endoscopic surgery. By taking advantage of the features of these scopes, we report the usefulness of the minimally invasive combined exoscopic and endoscopic two-step keyhole approach (EEKA) for various types of meningiomas. We reviewed data from 34 consecutive cases of EEKA for various types of intracranial meningiomas compared with that of conventional microsurgery. All of the tumors were resected as planned without severe complications. Significantly better outcome data were obtained in terms of the blood loss and the surgical time in the EEKA group, in addition to the craniotomy size. The well-illuminated fine vision in the deep corners by the endoscope enabled radical resection of the tumors with minimum burden on the patients. This technique has the potential for minimally invasive surgery in intracranial meningioma patients, including the older population.

## 1. Introduction

The advantages of neuroendoscopic surgery are the wide viewing angle and the freedom of an axis of view with minimal surgical trauma. A minimally invasive transcranial keyhole approach using an endoscope was reported by the pioneers in this field in the early 21st century [1,2,3]. Since 2007, we have been working on a minimally invasive purely endoscopic keyhole approach for various neurosurgeries, and we have devised surgical methods and have developed special tools. In surgery for intracranial meningiomas, observation beyond the corner—for example, in the optic canal, the olfactory groove, and the base of the middle fossa—via a minimum keyhole craniotomy is possible by using an endoscope. Surgical radicality is improved by reducing the blind spot when using an endoscope [4,5,6,7,8,9]. The fact that the entrance can be made smaller not only reduces the burden on the wound but also reduces the unnecessary brain retraction. For patients with meningioma, especially those patients who are older, what is most important is that the tumor does not cause lifelong symptoms, rather than a complete removal of the tumor. Accordingly, maximum internal decompression with an endoscope that does not expose the brain, especially in huge tumors with brain edema, may be reasonable in selected patients with meningioma who are older [10,11,12]. Although there have been reports of minimum invasive endoscopic approaches to meningiomas, there have been few reports and analyses of a series of cases. In addition, reports on the use of an exoscope, which is compatible with an endoscope, have been increasing in recent years [13,14]. We also recognized the advantages of an exoscope in our series, such as a small body, the freedom of the visual axis, good ergonomics, and a smooth conversion with the endoscope. By taking advantage of the features of these scopes, we report the usefulness of a minimally invasive combined exoscopic and endoscopic two-step keyhole approach (EEKA) for various meningiomas based on a historical comparison with conventional microsurgery.

## 2. Materials and Methods

In this study, data were collected from 34 consecutive cases with intracranial meningioma who underwent EEKA performed by the authors at the Japanese Red Cross Nagoya Daini Hospital and Aichi Medical University from July 2018 to June 2021 (EEKA group). During this period, all the cases of intracranial meningiomas underwent EEKA. The 2D exoscope (Storz VITOM 90 degree, Tuttlingen Germany) was used in the first 6 cases and the 3D exoscope (Storz VITOM 3D, Tuttlingen, Germany) was used in other cases; both of which have been combined with an endoscope.

In order to make a comparison with conventional microsurgery, we collected data from 30 consecutive cases of microsurgery for intracranial meningiomas, except convexity meningiomas with a diameter of 3 cm or less, that were performed at Aichi Medical University from January 2016 to June 2018 (Micro group). With regard to surgical indications, both groups were patients who presented with neurological symptoms due to the tumor or who showed an increase in follow-up imaging studies. As convexity meningiomas with a diameter of 3 cm or less were not included in the EEKA group, they were also excluded from the Micro group.

Patients’ age, gender, blood loss (mL), operation time (min), hospitalization period including rehabilitation (days), pathological diagnosis (WHO grade), Ki67 index (%), operative position, operation site (frontal base: FB, posterior fossa: PF, falcine: Falx, middle fossa: MF, ventricle: Vent, convexity: Convex), tumor size (major axis × minor axis × height/2 in cm^3^), craniotomy size (major axis × minor axis in cm^2^), neurological symptoms caused by the tumor, postoperative symptom changes, degree of excision (Simpson grade 1–4) [15], complications, presence of recurrence, and additional treatment were recorded in each case.

The amount of blood loss was recorded as 3 mL when the amount was too small to measure. The length of hospital stay included the period of rehabilitation. Details of the surgical site were as follows: FB includes olfactory groove, tuberculum sellae, and anterior clinoid; PF includes petro-clival, petro-tentorial, tentorial, torcular, and foramen magnum; Falx includes parasagittal and falx; MF includes middle fossa and sphenoid ridge; Vent includes trigon and velum interpositum.

Categorical variables were compared using the chi-squared tests, and continuous variables were compared using *t*-tests as univariable analyses. The general linear model was used to compare the amount of blood loss, operation time, craniotomy size, and length of hospital stay between the EEKA group and the Micro group, adjusted for age (continuous), gender, tumor size, preoperative symptoms (asymptomatic/symptomatic), operation site (PF/FB + MF/Falx + Vent + Convex), and operative position (Supine/Prone/PB). In the analyses, the amount of blood loss, operation time, craniotomy size, and length of hospital stay were natural-logarithmically transformed in order to approximately normalize their distributions. Statistical analyses were performed using SPSS 28.0 (IBM, Chicago, IL, USA).

### 2.1. Surgical Procedure for EEKA

**Figure 1:**
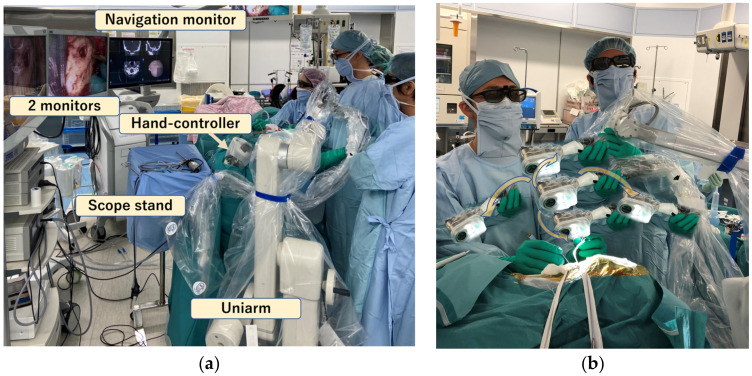

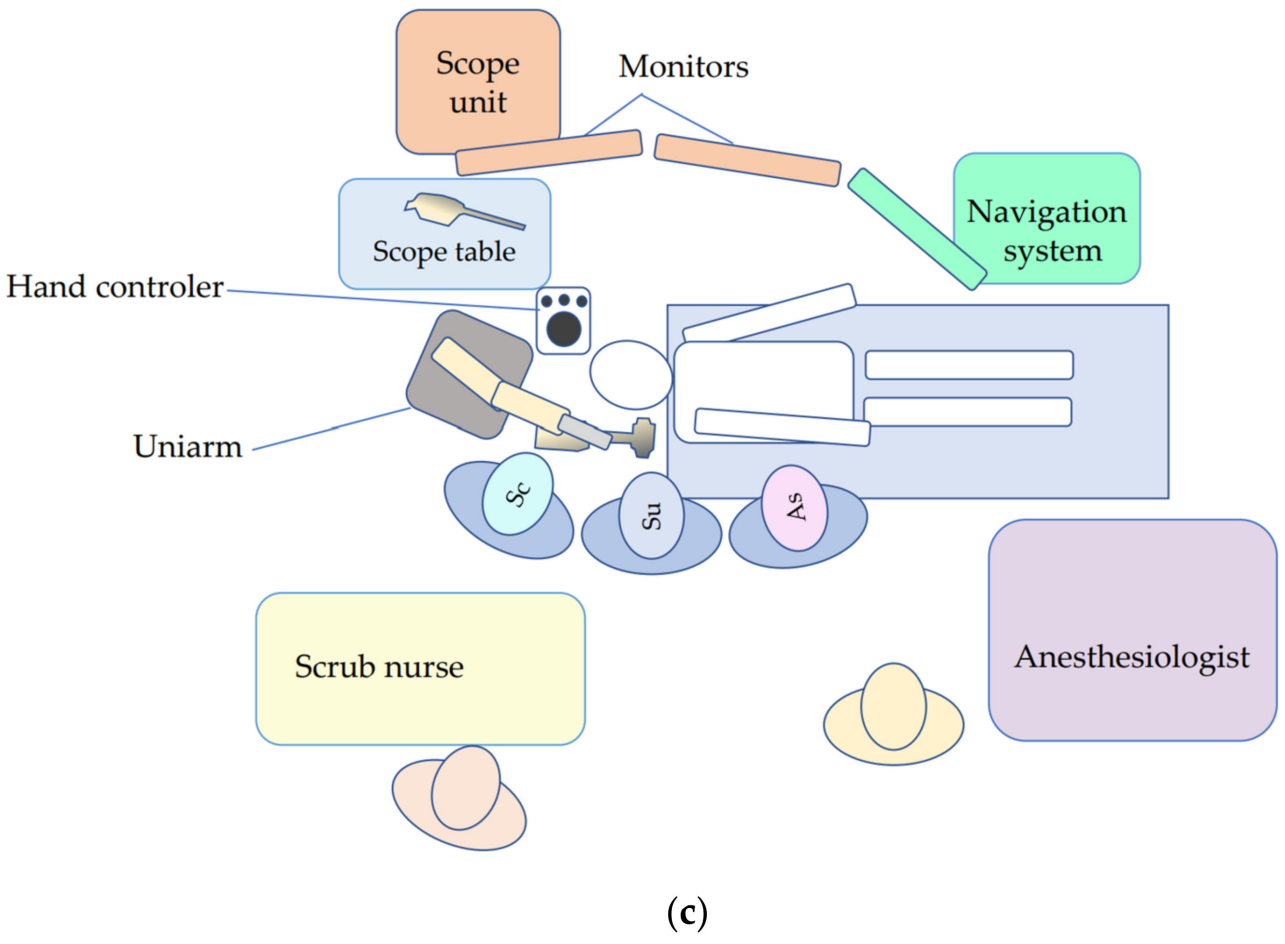
(**a**) Intraoperative photo of the lateral suboccipital approach. The exoscope is being used in this moment, while the endoscope is placed on the scope table. (**b**) The scopist repositions the exoscope as appropriate to maintain the best field of view. Regardless of the movement of the scope, the surgeon can operate with a certain comfortable posture. The four additional processed exoscope photographs were created to represent its movement. (**c**) The layout of operation room. Sc: scopist, Su: surgeon, As: assistant.

**Figure 2:**
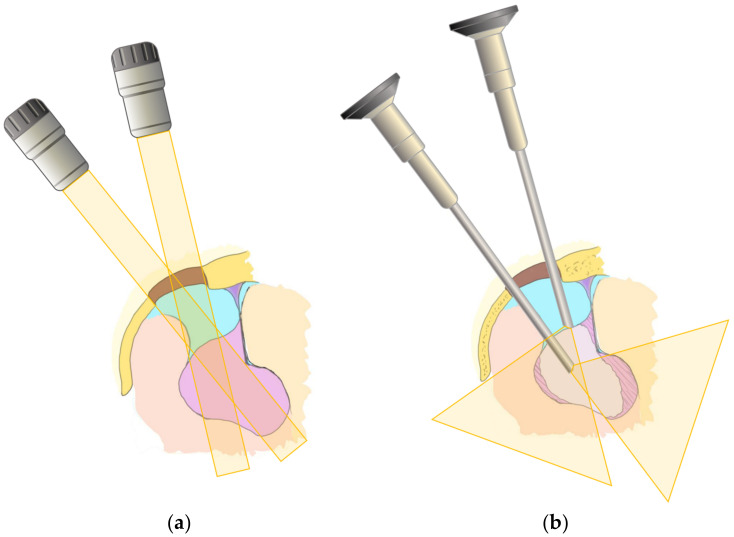
Scheme of observation by exoscope as the first step (**a**), and by endoscope as the second step (**b**).

**Figure 3:**
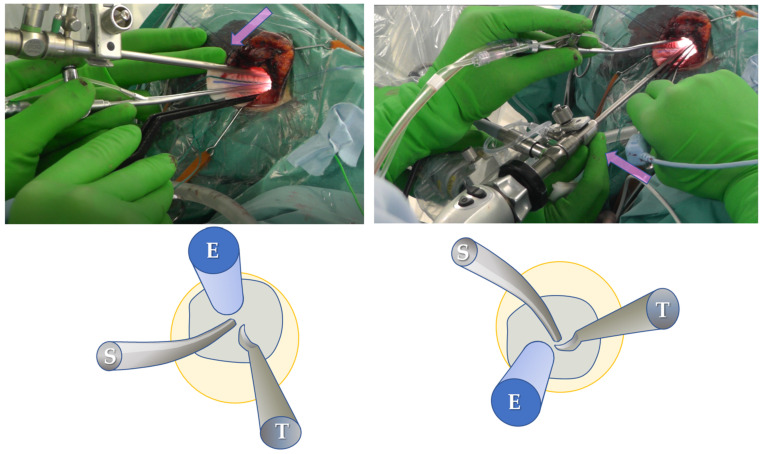
Photographs of the endoscopic keyhole approach and schemas showing the position relationship of the endoscope (E), suction (S), and tool (T). The craniotomy is represented by the blue line and the yellow circle indicates the field of view of the endoscope. The shaft of the endoscope is placed on the craniotomy edge, and delicate control is possible by touching it with a finger, similar to supporting a billiard cue.

In all cases, meticulous preoperative planning was carried out based on the anatomical considerations for determining the site, size, and optimal placement of the craniotomy, as well as the trajectory toward the surgical target. Under general anesthesia, the patient’s head was fixed in a frame, and tumor removal was performed using an endoscope (Storz Image 1 High definition/4K, Tuttlingen, Germany) and an exoscope (Storz VITOM 2D/3D, Tuttlingen, Germany). The scopes were held by a floor-standing pneumatic holder (Uniarm Mitaka, Tokyo, Japan) that was controlled by the assistant (scopist) to continue the procedure in a comfortable environment. The scopist controlled the position of the scope to provide a clear and detailed observation. The zoom and focus of the exoscope were also controlled by the scopist, using the hand-controller fixed on the bedrail (Figure 1). The surgeon was able to continue the operation with two hands without stopping because the scopist moved the exoscope appropriately every moment, captured the surgical field, and adjusted the zoom and focus. When necessary, verbal communication assisted the changing of the surgical fields or subtle angles. As a first step, surgery was performed with the exoscope until the tumor was detached, devascularized, and decompressed to the greatest extent possible with a small craniotomy. The scope was then changed to an endoscope to observe regions that were difficult to see from the keyhole, using the exoscope as a second step (Figure 2). With the endoscope attached to the holder, the surgeons could continue the procedure by looking at the same monitor without changing their posture. The scopist controlled the position of the endoscope according to the operation of the surgeon, avoiding interference with the surgeon’s tools. Surgical tools were placed on the other side of the endoscope (Figure 3). As the endoscope was located inside the skull and was surrounded by fragile structures, delicate manipulations were required. Therefore, the scopist fixed the endoscope shaft against the edge of the craniotomy and then placed their finger against the shaft to enable subtle movements, similar to holding a billiard cue (Figure 3). The surgeon, scopist, and another assistant stood almost side by side, and high-definition monitors were placed in front of them, while the scope table was placed near to the console of the endoscope system, where the unused scope was stored (Figure 1c).

### 2.2. Selection of Surgical Tools for EEKA

**Figure 4:**
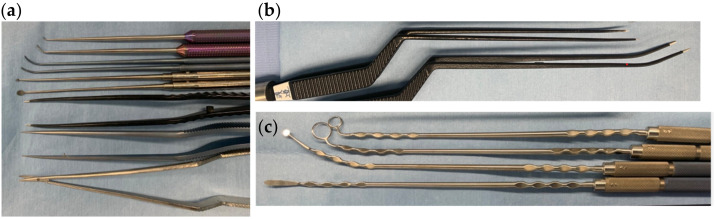
Our surgical tools for EEKA. (**a**) Conventional microsurgical instruments and dissectors with a curved tip. (**b**) Thin and long malleable bipolar forceps (FUJITA Medical Instruments Co., Ltd., Tokyo, Japan). (**c**) Malleable dissectors (Mizuho Medical Co., Ltd., Tokyo, Japan).

The selection of surgical tools used to safely perform this procedure is also important. Ordinary microsurgical fine tools can be used in manipulation with an exoscope. The tools with a slightly curved tip are useful in endoscopic surgery (Figure 4a). In particular, when the endoscope is placed close to the object, or when an angled endoscope is used, curved or malleable tools are required to avoid collision with the endoscope. Thin and long malleable bipolar forceps are useful (Figure 4b. FUJITA Medical Instruments Co., Ltd., Tokyo, Japan). The shape of the tip is repeatedly finely adjusted according to the part that is to be coagulated and the position of the endoscope. For the same reason, dissectors that can bend vertically and horizontally to make fine adjustment are also useful (Figure 4c. Mizuho Medical Co., Ltd., Tokyo, Japan). We have developed dissectors that can bend in two places, the tip and the handle. By bending as much as is necessary, it is possible to avoid collision with an endoscope in the surgical field or a camera outside of the surgical field. This bendable part is tough enough to withstand the force of the dissection and has a wave design for easy reshape. There are many variations in the shape of the tip.

### 2.3. Frontal Base Meningiomas

A supraorbital approach with a 5 cm eyebrow incision along the superior edge of the eyebrow was selected in most cases. A few patients preferred minimum hemi-coronal skin incisions for cosmetic reasons. The patient’s head was fixed in a head holder that was rotated slightly to the contralateral side of the tumor. In cases of large clinoidal meningiomas, 5 cm frontotemporal craniotomies were performed to achieve the anterior clinoidectomy and elevation of the lateral wall of the cavernous sinus that included the attachment was performed to control the feeding arteries from the skull base. Otherwise, a ~2–3 cm craniotomy was performed.

### 2.4. Middle Fossa Meningiomas

In supine position, the patient’s head was fixed with a rotation of 60–70°. A curved 5 cm skin incision was made above the ear without mobilization of the zygomatic arch, and a ~3 cm craniotomy was performed.

### 2.5. Posterior Fossa Meningiomas

In supine lateral position, the patient’s head was rotated 90° and fixed, and a 2–3 cm keyhole was made to achieve the lateral suboccipital approach with a 6 cm curved skin incision. Using an exoscope, it was possible to maintain an ergonomically comfortable posture for the surgeon even when observing with the horizontal visual axis at the approach stage. For midline lesions, such as tentorial meningiomas, torcular meningiomas, and foramen magnum meningiomas, a prone position was typically selected.

### 2.6. Falcine and Convexity Meningiomas

**Figure 5:**
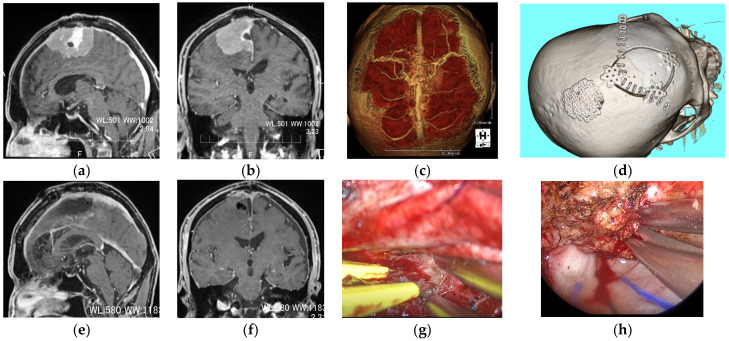
An 83-year-old male patient complaining of left lower-limb weakness underwent endoscopic intratumoral decompression and detachment of the tumor from the falx 1 year prior, after which the symptoms improved; however, because of its rapid growth, total resection of the tumor was planned. (**a**,**b**) Preoperative contrast-enhanced T1-weighted images (CET1WI sagittal and coronal). (**c**) A 3D reconstructed image of enhanced MRI showing diploic veins and bridging veins. Since the diploic vein may be involved in cerebral perfusion, and there was also a large bridging vein just above the tumor, it was planned to remove the tumor of the parasagittal sinus from the frontal side under endoscopic observation. The craniotomy was designed to be 6 cm in length and 4 cm in width anterior to the bridging vein, with his head rotated 80° to the ipsilateral side and fixed as shown in (**d**). Dissection of strong adhesions to the brain and internal decompression were performed using an exoscope (**g**). The observation and dissection at the posterior site and parasagittal attachment were performed using an endoscope (**h**). (**e**,**f**) Postoperative CET1WI sagittal and coronal view showing gross total resection of the tumor.

In supine position, the patient’s head was fixed with a rotation of 70–80° with the ipsilateral side down, expecting natural brain retraction due to gravity in cases of falcine meningiomas. In cases of falcine meningiomas that were concealed by the developed bridging veins or diploic veins, a slightly elongated craniotomy was made anterior to the lesion, and an interhemispheric fissure was used for an endoscopic approach (Figure 5). In the case of a huge en plaque meningioma in a patient who was older, a 4 cm × 6 cm craniotomy was made at the center of the attachment. Maximum internal decompression was achieved using an exoscope and endoscope, leaving a thin layer of tumor on the brain surface to avoid brain damage [10].

The Institutional Review Board approved this retrospective clinical study (approval number 2021-118).

## 3. Results

### 3.1. Patient Characteristics

The EEKA was performed in 34 patients whose mean age was 62.4 years old, including 10 male and 24 female patients. The operation sites were as follows: frontal skull base lesions (FB, *n* = 5), posterior fossa lesions (PF, *n* = 17), falcine lesions (Falx, *n* = 5), ventricle lesions (Vent, *n* = 3), middle fossa lesions (MF, *n* = 2), and convexity lesions (Convex, *n* = 2). The surgeries were performed in a supine position (*n* = 26), a prone position (*n* = 8), or a lateral position (*n* = 0). The mean tumor size was 17.3 ± 14.8 mL. The preoperative symptoms due to the tumor were confirmed in 18 cases, including visual dysfunction (*n* = 5), motor weakness (*n* = 2), gait disturbance (*n* = 3), psychiatric symptoms (*n* = 3), trigeminal neuralgia (*n* = 3), trigeminal neuropathy (*n* = 1), and hoarseness (*n* = 1), whereas the remaining 16 cases were asymptomatic.

The data were obtained from 30 patients in the Micro group whose mean age was 59.5 years old, including 8 male and 22 female patients. The mean tumor size was 19.2 ± 20.6 mL. There was no significant difference between the two groups in terms of age and tumor size. Other data are listed in Table 1, in comparison with the EEKA group.

### 3.2. Postoperative Evaluation

**Figure 6:**
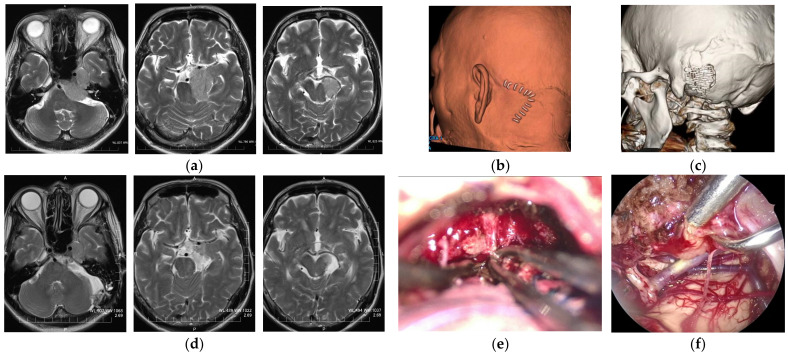
A 60-year-old male patient who complained of gait disturbance due to compression of the brain stem. (**a**) Axial T2-weighted images showing a right petro-clival meningioma. (**b**,**c**) Postoperative 3D CT images presenting a curved skin incision and retro-mastoid craniotomy fixed by a titanium plate. In this case, where the combined petrosal approach or staged surgery would have been selected in conventional microsurgery to achieve maximum resection, the tumor was partially removed by a lateral suboccipital approach using an exoscope (**e**), and the tumor in the ambient cistern that compressed the midbrain was removed by endoscopic observation (**f**). (**d**) Postoperative axial T2-weighted images showing the tumor removed, except the part in the cavernous sinus, while the compression of the brain stem was released.

In the EEKA group, all of the tumors were resected as planned, with minimum brain retraction and well-illuminated fine vision in the deep corners. The representative cases are shown in Figure 5 and Figure 6. The mean blood loss was 120.7 ± 123.6 mL and the mean surgical time was 256.8 ± 109.9 min. The mean hospital stay, including the rehabilitation period, was 15.9 ± 9.5 days. The mean craniotomy size (the product of the major and minor axes in cm^2^) was 9.4 ± 7.1 cm^2^. The mean Ki67 index was 3.3 ± 2.8%. When compared with the Micro group, there were significant differences in blood loss, surgical time, hospital stay, and craniotomy size (even after adjusting for the operation site), operative position, and tumor size, which showed the minimal invasiveness of the EEKA group (Table 2).

The preoperative neurological symptoms that were due to the tumor were improved in thirteen cases and no change was observed in five cases. None of the fourteen asymptomatic patients developed any new postoperative neurological symptoms. Regarding the extent of the tumor resection, six cases were Simpson grade one, eighteen cases were Simpson grade two, two cases were Simpson grade three, and eight cases were Simpson grade four. There were nine cases who were aged 75 years or older, seven of whom had tumor symptoms, and six of whom showed improvement. Four of the nine patients who were older were Simpson grade two, and five patients were Simpson grade four. In all of the cases that resulted in partial removal, the removal was intentionally limited so as not to exacerbate the symptoms.

The complications in both of the groups are shown in the Table 3. All of the cases of cranial nerve palsy recovered completely within 2–3 months after surgery.

Postoperative recurrence occurred in three cases in the EEKA group, including one case of atypical meningioma. In the case of a patient who was older and who underwent intentional partial excision, total resection was performed one year later due to regrowth. In one case of anterior clinoidal meningioma, a subtle remnant of the tumor remained in the optic canal, and reoperation was performed, due to visual symptoms, two years after the initial surgery. In a case of falcine atypical meningioma, which had a tumor recurrence away from the surgical site, it was controlled by SRS at the recurrent site. Of the three recurrent cases, two cases were WHO grade one, with a high Ki67 index over 5% [16].

## 4. Discussion

By taking advantage of the wide viewing angle of the endoscope, even when using a small keyhole, the blind spot was significantly reduced and highly curative extraction was achieved. Except for the cases of minimally invasive surgery for the patients who were older and those with partial removal in order to preserve brain stem function, the removal rate was Simpson grade two or higher and all of the cases were removed as planned, with no mortality or serious complications. There were nine cases who were aged 75 years or older, seven of whom had tumor symptoms, and six of whom showed improvement. Regarding the older population, the minimum necessary treatment was performed through a small keyhole in order to avoid worsening of symptoms, and, as a result, the symptom improvement rate was 85.7%, which is higher than the overall symptom improvement rate. It is important that the surgery is completed with minimal invasiveness, with necessary and sufficient removal. In the aging society in the future, meningioma is considered to be a benign condition that can coexist without disturbing the quality of life in many cases. The role of the endoscope, which achieves a minimally invasive approach, is considered to be important for the older population. However, it should be remembered that some older meningioma patients have malignant meningiomas and fast-growing tumors, therefore, necessitating a re-operation for total excision [17].

In the EEKA group, the craniotomy size was significantly smaller compared to the conventional approach using a microscope. In many cases, deep-seated tumors around the corner could be removed via a small craniotomy, and the strategy was simplified. A petro-clival meningioma, which previously required a combined petrosal approach, was fully addressed by using a retro-sigmoid approach. The clinoidal meningiomas and middle fossa meningiomas, which previously required zygomatic osteotomies, could be adequately dealt with by only using a small craniotomy, while omitting the need for bone removal. A craniotomy size of 2 to 3 cm not only reduces the burden on the skin and skull, but also potentially protects against the unnecessary retraction of the brain. In addition, since the endoscope itself and the instrument stabilize upon touching the craniotomy edge, this is an appropriate size in terms of fine control of the endoscope and the tools.

There was a significant difference in blood loss, surgical time, and hospital stay in addition to the craniotomy size between the two groups, even though there was no difference in the tumor size. The minimal invasiveness of this method was shown in this study. Regarding the surgical time, besides the absolute difference in the size of the wound, it is considered that the scopist moving the scope appropriately saves time for the operator to interrupt the two-hand manipulation and contributes the shortening of the surgical time.

The exoscope is compatible with the endoscope, and, by adding it to endoscopic surgery, a seamless and optimal environment can be obtained. With the advent of the exoscope, it is possible to shift to the endoscope at any time during a heads-up surgery that is performed by looking at a monitor, and the viewpoint can be freely moved between the outside and the inside. Furthermore, since the visual axis is free, the surgery can be continued in an ergonomically comfortable posture for both the surgeons and the patients. Therefore, a retro-sigmoid approach was possible with supine lateral position, and there were no complications, such as pressure ulcers or pain, that were associated with the position. In the falcine lesions, it was possible to use gravity-based traction of the brain, and, therefore, it was possible to perform the surgery on the horizontal or looking-up axis without physical stress for the surgeons. In addition, the scopist can move the scope and adjust the focus and zoom, so that the surgeon can continue the surgery from the best viewpoint without interrupting the two-hand manipulation. For that reason, the scopist needs to understand the surgery and must proceed with the surgery in harmony with the surgeon. This method takes full advantage of the keyhole concept, which moves the viewpoint more and more as needed, but the surgeon does not have to move their body accordingly; instead, they can continue looking at the monitor in front of them (Figure 1b). Nonetheless, there is a physical blind spot; thus, it is reasonable to change to an endoscope when the keyhole reaches an area that cannot be seen with an exoscope. The conversion from an exoscope to an endoscope can be performed quickly, and the surgeons can continue the surgery without changing their posture.

The collision of instruments, which is a drawback of endoscopic manipulation, was solved through the cooperation between the scopist and the surgeon and the selection of appropriate surgical instruments. The scopist’s method of controlling the position, the focus, and the zoom of the scope is important in this surgery, especially during the operation with the endoscope, because collision with the endoscope body is always a problem. The surgeon can obtain safety by always adjusting the scope to the appropriate position. If the scopist is familiar with endoscopic operation, the endoscope can be placed at a point where the surgeon can observe in detail without disturbing the operation. In addition, since malleable tools, such as suction tips and dissectors, are effective in the surgical field when the endoscope is close, it is also essential to prepare the tools that are suitable for the endoscopic procedure in advance.

### Limitations

This study targeted a variety of meningiomas and was not a prospective study. It featured a limited number of cases from two institutions and had a limited follow-up period. It is undeniable that the relatively large number of posterior fossa lesions in the EEKA group may have influenced the results of the surgical invasiveness comparison. It cannot be said that there was a clear and significant minimal invasiveness.

Among the five cases of tuberculum sellae and clinoidal meningioma, there were two cases in which an anterior clinoidectomy and optic nerve decompression were performed on the basis of feeder control and tumor removal in the optic canal. Although the zygomatic osteotomy could be omitted, the craniotomy was 4 cm × 5 cm, which was the same size as the conventional craniotomy. In the cases of this series, the anterior clinoidectomy and the unroofing of the optic canal were determined by the degree of tumor invasion into the optic canal during the preoperative examination. It is difficult to predict the pathological diagnosis or to determine the presence or absence of microtumors in the optic nerve canal from the preoperative MRI images alone. Therefore, there was a limitation in appropriately deciding a minimally invasive surgical strategy.

This procedure allows the surgeon and the scopist to perform the surgery together, similar to the relationship between the neurosurgeon and the otolaryngologist, who is the scopist during endoscopic endonasal neurosurgery using a four-hand technique. It can be said that this style requires an assistant who is familiar with brain tumor surgery and endoscopic surgery, in addition to the surgeon. This point may be a disadvantage; however, in terms of surgeon education, it can be said that it has educational value because the scopist plays an important part in the surgery. On the other hand, it is also possible to guide the surgeon if the experienced leading surgeon plays the role of the scopist. Although some experience is required in order to become familiar with endoscopic manipulation in order to perfect this technique, with the scopist’s support, the learning curve can be shortened.

## 5. Conclusions

The combined exoscopic and endoscopic two-step keyhole approach for meningioma surgery is a minimally invasive approach that features a wide viewing angle, which allows for radical removal of the tumor. This approach provides a comfortable posture for both the surgeon and the patient, due to the high degree of freedom of the visual axis. The scopist’s delicate control of the scopes allows the surgeon to concentrate on the surgery with minimal instrumental interference. This technique has the potential for minimally invasive surgery in intracranial meningioma patients, including those in the older population.

## Figures and Tables

**Table 1:** Patient characteristics.

	Micro	EEKA	*p* Value
Total number	30	34	
Male:Female	8:22	10:24	0.81
Mean Age	59.5 ± 14.4	62.4 ± 15.5	0.44
Mean tumor size (mL)	19.2 ± 20.6	17.3 ± 14.8	0.68
Operation site	*n*	*n*	0.08
PF	9 (30.0%)	17 (50.0%)	
FB/MF	8/6 (46.7%)	5/2 (20.6%)	
Falx/Vent/Convex	4/1/2 (23.3%)	5/3/2 (29.4%)	
Operative position	*n*	*n*	<0.01
Supine	21 (70.0%)	26 (76.5%)	
Prone	2 (6.7%)	8 (23.5%)	
Lateral	7 (23.3%)	0 (%)	
Preoperative symptoms	*n*	*n*	0.44
Asymptomatic	17 (52.9%)	16 (43.3%)	
Symptomatic	13 (47.1%)	18 (56.7%)	
Visual dysfunction	1	5	
Motor weakness	2	2	
Gait disturbance	2	3	
Psychiatric symptoms	5	3	
Trigeminal neuralgia	1	3	
Trigeminal neuropathy	0	1	
Hoarseness	0	1	
Hearing disturbance	1	0	
Headache	1	0	

**Table 2:** Post-operative evaluation.

	Micro	EEKA	*p* Value	Adjusted *p* Value
Mean blood loss (mL)	417.8 ± 361.0	120.7 ± 123.6	<0.01	<0.01
Mean surgical time (min)	364.2 ± 143.8	256.8 ± 109.9	<0.01	<0.01
Mean hospital stay (days)	23.5 ± 12.4	15.9 ± 9.5	0.01	<0.01
Mean craniotomy size (cm^2^)	35.0 ± 17.5	9.4 ± 7.1	<0.01	<0.01
Mean Ki67 index (%)	2.5 ± 1.9	3.3 ± 2.8	0.19	NA
Simpson grade			0.25	NA
1	7 (23.3%)	6 (17.6%)		
2	17 (56.7%)	18 (52.9%)		
3	4 (13.3%)	2 (5.9%)		
4	2 (6.7%)	8 (23.5%)		
Postoperative symptoms change	*n* (total 13)	*n* (total 18)	0.77	NA
Improved	10 (76.9%)	13 (72.2%)		
No change	3 (23.1%)	5 (27.8%)		
Worsened	0 (%)	0 (%)		
Simpson grade (>75 years old)	*n* (total 4)	*n* (total 9)	0.24	NA
1	0 (%)	0 (%)		
2	2 (50.0%)	4 (44.4%)		
3	1 (25.0%)	0 (%)		
4	1 (25.0%)	5 (55.6%)		
Postoperative symptoms change (>75 years old)	*n* (total 3)	*n* (total 7)	0.10	NA
Improved	1 (33.3%)	6 (85.7%)		
No change	2 (66.7%)	1 (14.3%)		
Worsened	0 (%)	0 (%)		

**Table 3:** Postoperative complications.

Complications	Micro *n* = 13	EEKA *n* = 5
Transient cranial nerve palsy	4	4
Transient attention disorder	1	1
Cerebral infarction	1	0
Venous infarction	2	0
Transient sensory disturbance	1	0
Smelling disorder	2	0
Poor wound healing	1	0
Postoperative hemorrhage	1	0

## Data Availability

The data presented in this study are available on request from the corresponding author.
